# Robson’s Group 2 Criteria among Total Caesarean Sections in a Tertiary Care Hospital: A Descriptive Cross-sectional Study

**DOI:** 10.31729/jnma.7138

**Published:** 2021-11-30

**Authors:** Pratigyan Gautam, Chanda Karki, Asmita Adhikari

**Affiliations:** 1 Department of Obstetrics and Gynaecology, Kathmandu Medical College and Teaching Hospital, Sinamangal, Kathmandu, Nepal

**Keywords:** *caesarean section*, *Nepal*, *Robson's criteria*

## Abstract

**Introduction:**

Globally, there is a dramatic rise in cesarean section rate which has increased the maternal morbidity and adverse effects in a subsequent pregnancy. Robson's classification will aid in the optimization of the cesarean section use, assessment of the strategies aimed to decrease the cesarean section rate and thus improve the clinical practices and quality of care in various health care facilities. The main aim of this study is to find out the prevalence of caesarean section for Robson's group 2 among total caesarean sections done in a tertiary care hospital.

**Methods:**

A descriptive cross-sectional study was carried out at a tertiary care centre in Nepal from August 2020 to January 2021. Ethical approval was taken from the institutional review committee (reference number: 1607202003) and data were entered using Robson's criteria. The convenient sampling technique was used. Data was analyzed using Microsoft Excel. Point estimate at 95% Confidence Interval was calculated along with frequency and percentage for binary data.

**Results:**

According to Robson's group 10 classification, among 380 caesarean sections, 110 (28.94%) (24.38-33.50 at 95% Confidence Interval) cases were in Robson's group number 2.

**Conclusions:**

This study showed that the prevalence of caesarean section which lies in Robson's group 2 in our study is higher than the standard of World Health Organization. It showed that Robson's group 2 was one of the significant contributors to the greater caesarean section rate. Improved case selection, standardization, and protocol for induction of labour as well as regular audit could also reduce caesarean section rates.

## INTRODUCTION

Caesarean section (CS) is a lifesaving surgical procedure for delivering the baby when complications arise either to the mother or fetus.^1^ According to the World Health Organization (WHO), CS ranges from 10-15%.^2-5^ There is a dramatic rise in CS rate globally, which has increased maternal morbidity and it caused an adverse effect in subsequent pregnancy such as abortions, stillbirths, placenta previa, retained placenta, postpartum haemorrhage, and infections.^6,7^

According to the latest data from 150 countries, currently, 18.6% of all births occur by CS.^1^ The CS rate in Nepal was 9% in 2016, 5.9% in rural and 11.7% in urban.^7,8^ According to WHO, Robson's classification will aid in the optimization of CS use, assessment of the strategies aimed to decrease the CS rate and thus improve the clinical practices and quality of care in various health care facilities.^9^

The main aim of this study is to find out the prevalence of Robson's group 2 among total caesarean sections done in a tertiary care hospital.

## METHODS

This was a descriptive cross-sectional study conducted in the Obstetrics and Gynaecology Department of Kathmandu Medical College. The study was done over six months (from August 2020 to January 2021) after taking ethical clearance from the Institutional Review Committee of Kathmandu Medical College (Reference number: 1607202003). Pregnant ladies who were planned for CS were included in the study. Incomplete data in the record was excluded. A convenience sampling technique was done. The sample size was calculated using the formula:

n = Z^2^ × p × q / e^2^

  = (1.96)^2^ × 0.5 × (1-0.5) / (0.06)^2^

  = 267

where,

n= required sample size,
Z= 1.96 for 95% Confidence Interval (CI),
p= 50%, for maximum sample size calculation
q= 1-p
e= margin of error, 6%

The calculated minimum sample size was 267. However, the total sample size taken was 380.

The data were collected from the hospital records of the deliveries done in the Department of Obstetrics and Gynaecology of Kathmandu Medical College from August 2020 to January 2021. Demographic profile and all relevant parameters like obstetric history, the onset of labour, fetal lie, number of neonates, and gestational age were collected from the record. Based on those parameters, cases were categorized as one of Robson's criteria.^10^ The criteria consist of 10 groups ranging from 1 to 10 and has been appreciated by WHO in 2014 and FIGO in 2016.^9,11^

It classifies all deliveries into one of ten groups based on five parameters: obstetric history (parity and previous caesarean section), the onset of labour (spontaneous, induced, or caesarean section before onset of labour), fetal presentation or lie (cephalic, breech, or transverse), number of neonates, and gestational age.^12^

Data was analyzed using Microsoft Excel. Point estimate at 95% Confidence Interval was calculated along with frequency and percentage for binary data.

## RESULTS

**Figure 1:**
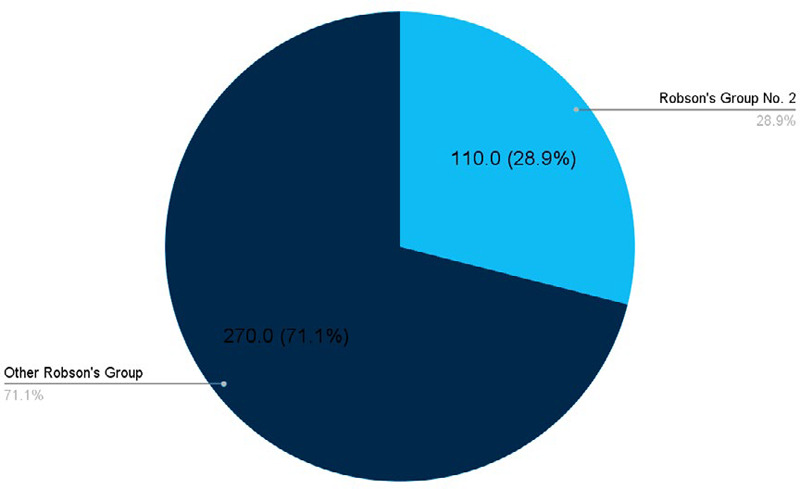
Robson's group number 2 among total cesarean sections (n= 380).

According to Robson's group 10 classification, among 380 caesarean sections, 110 (28.94%) (24.38-33.50 at 95% Confidence Interval) cases are in Robson's group number 2 (nulliparous, singleton, cephalic, ≥37 weeks, induced labour or cesarean section before labour) (Figure 1).

Among the study population of 380 ladies, the mean age of the population was found to be 28.3 years±4.41. The maximum of those ladies was nulliparous which was found to be 187 (49.2%) out of the total study group. The majority of the cases were done at term pregnancy, which accounts to be 342 (90%) (Table 1).

**Table 1:** Age, parity, period of gestation of women undergoing caesarean section (n= 380).

Variables	Frequency n (%)
**Age Group**	
15-19 years	5 (1.31)
20-24 years	74 (19.47)
25-29 years	156 (41)
30-34 years	108 (28.42)
35-39 years	36 (9.47)
40-44 years	1 (0.26)
**Parity**	
0	187 (49.21)
1	178 (46.84)
2	12 (3.15)
3	3 (0.78)
**Period of gestation**	
< 37 weeks ≥ 37 weeks	38 (10) 342 (90)

## DISCUSSION

Robson's criteria were used for the first time in our department for the classification of caesarean section, and it had helped us to find out the most common indication for caesarean section. Our study showed the rate of caesarean section in our hospital was found to be 48.28% which is quite high compared to WHO criteria (15%).1n 2016, a study was done by the ministry of health of Nepal has shown that the CS rate in Nepal is 9%. In this study, a major contribution for caesarean section was found to be from group 2 and this shows that we need to be more attentive and concerned regarding proper indications and methods of induction of labour.

A study done by Poudel R, et al. at Kathmandu Model Hospital showed overall caesarean section rate was 66.1% (494 among 747 total deliveries) in 2018.^13^ Nulliparous, singleton cephalic, ≥37 weeks, spontaneous labour (Group 1) was the major (24.2%) contributor to the overall caesarean section rate.

Malla RV, et al. did a retrospective study at Shree Birendra Hospital, over five years from August 13, 2012, to August 11, 2017.^14^ A total number of 4892 deliveries were conducted over the five-year study period. Robson's Group 1 was the highest contributor to the overall CS rate, contributing 28% of all C-sections, followed by Group 5 (26.8%) and Group 3 (15.5%).

Similarly, Gautam B, et al. did a retrospective crosssectional study from 2016 April -2017 March in Lumbini Zonal Hospital, Butwal, Rupandehi, Nepal.^15^ Total of 3,817 women who gave birth over 12 months were analyzed using this classification. Women with previous CS (Group 5) comprise the largest proportion (9.4%) of the overall 26.41% CS rate.

In India, Kant A, et al. did a study over 6 months and found out that there was a trend of an increased percentage of cesarean section in group 5 (multiparous with prior cesarean section, singleton, cephalic, ≥37 weeks) and 2 (nulliparous, singleton, cephalic, ≥37 weeks, induced labour or cesarean section before labour) which was 36 and 36.71 percent respectively.^3^

Likewise, another study was done in India by Yadav RG, et al. over 10 years from 2004 to 2013, the 10-year overall cesarean section rate (CSR) was 25.17 %. The largest contributions to the total caesarean section rate were found to be group 1 (37.62 %).^16^

Missing data could bias the estimation of the outcome of interest and also might reduce the representativeness of the sample. The choice of convenience sampling introduces the potential selection bias. We cannot generalize the results to the target population due to the under-representativeness of the subgroups in the sample in comparison to the population of interest. The present study had a relatively small sample size and was a cross-sectional study which limits the collection of data at a point in time.

## CONCLUSIONS

This study showed that the prevalence of caesarean section which lies in Robson's group 2 in our study is higher than the standard of WHO. Failed induction and previous CS were the underlying indications for performing CS. To minimize or optimize the CS rate, one needs to regularly check the indications of induction of labour and indications of CS. We might continue a few other good multicentric studies on indications and methods of induction of labour in different private and public institutions and recommend the recommendations to concerned authorities. Proper and honest indications of induction of labour and CS will certainly bring down the current high CS rate. Caesarean Section is a lifesaving procedure but needs to be done for truly indicated cases correctly only weighing its risk and benefit. This might be the reason why WHO has set the expected caesarean rate. This small study was done to see where we stand in this context.
